# Why is the Liverpool care pathway used for some dying cancer patients and not others? Healthcare professionals’ perspectives

**DOI:** 10.1186/1756-0500-5-524

**Published:** 2012-09-24

**Authors:** Alison Freemantle, Jane Seymour

**Affiliations:** 1 Hayward House, Nottingham University Hospitals NHS Trust, Hucknall Road, Nottingham, NG5 1 PB, UK; 2 Sue Ryder Care Centre for the Study of Supportive, Palliative and End of Life Care, University of Nottingham, School of Nursing, Midwifery and Physiotherapy, Queen’s Medical Centre, Derby Road, Nottingham NG7 2HA, UK

**Keywords:** Liverpool care pathway, End-of-life care, Hospital, Qualitative research, Cancer, Healthcare professionals

## Abstract

**Background:**

Despite evidence suggesting that the Liverpool Care Pathway for the Dying Patient is a structured and proficient means of supporting care delivery in the last hours or days of life, discrepancies in uptake are widespread. This exploratory study sought to understand why patients dying of cancer in oncology wards of one hospital trust were, or were not, supported by the LCP. A purposive qualitative case study design was used; each case represented a patient who had died and their most involved nurse and doctor. In-depth interviews explored both recollections of the ‘case’ and wider experiences of using the Pathway in end-of-life care. Eleven healthcare professionals were interviewed about their involvement in the end-of-life care of six patients. For four of these patients care was supported by the LCP.

**Findings:**

Although doctors and nurses reported they preferred to use the Pathway to ensure comfortable death, an important factor influencing their decisions was time of death. Access to timely senior review was regarded as an essential preliminary to placing patients on the Pathway but delayed access ‘out of hours’ was commonly experienced and tensions arose from balancing conflicting priorities. Consequently, the needs of dying patients sometimes failed to compete with those receiving curative treatment.

**Conclusions:**

This study suggests that greater attention should be focused on ‘out of hours’ care in hospitals to ensure regular senior review of all patients at risk of dying and to support front line staff to communicate effectively and make contingency plans focused on patients’ best interests.

## Findings

### Introduction

Most cancer patients die in hospitals [1]. The Liverpool Care Pathway for the Dying Patient (LCP) was initially introduced in the hospital setting to provide a framework for practitioners to improve end of life care, specifically the last few days to hours of life. Based on integrated care pathway methodology, the LCP was developed in the mid-1990s by Professor Ellershaw and his team in Liverpool to provide a comprehensive template of appropriate, evidence-based, multidisciplinary care for the last days of life [2]. The document defines 18 goals considered essential for optimal care of the dying; these include initial assessment and care, ongoing care and care after death. It provides guidance on anticipatory prescribing of medications, discontinuation of inappropriate interventions, comfort measures, psychological and spiritual care as well as communication with and support to the family both during and after death.

Evidence from qualitative and quasi-experimental studies suggests the LCP is a structured and proficient means of care delivery with some favourable outcomes [3-9]. The LCP is recommended for practice in England and Wales by the National Institute of Clinical Excellence [10] and has been adopted in 21 other countries [11].

### Current evidence base

Audit has been the main method of evaluation of the LCP with favourable outcomes reported including: significant improvement in documentation and improved access to effective measures for symptom control [12-16]. The results from: qualitative studies [3-6], a survey of bereaved relatives [7] and experimental assessments in non-randomised trials [8,9] suggest that the LCP can significantly improve the quality of end-of-life care delivery in hospitals by changing the emphasis of care, directing end–of-life decision making and providing a greater emphasis on the patient’s and family’s needs in care.

### Context of study

A locally adapted version of the LCP was implemented in a UK acute hospital Trust using a systematic roll-out programme. Adaptation included: name change to ‘Last Days of Life Pathway’ as this was thought to clearly identify the purpose of the framework, and the inclusion of flow charts to supplement the prescribing guidelines to enhance clarity. Additionally, the prescribing guidelines in the LCP were adjusted to reflect local practice. Education to underpin implementation was delivered in small group training sessions on the wards to nursing and medical teams by a full-time facilitator with support from the Hospital Palliative Care Team. Initial implementation across two large teaching hospitals took two years.

This small exploratory study took place before the current version of the LCP, version 12, was in use. At the time of the study, the LCP was used when the clinical team agreed that the patient was dying and where two of the following four criteria could apply: the patient was (i) bedbound, (ii) semi-comatose, (iii) only able to take sips of fluid and (iv) was no longer able to take tablets [2].

The driver for this study was that six years post implementation of the LCP, an internal baseline review of deaths on the three oncology wards in the hospital Trust showed that only 66% of cancer patients with advanced disease whose deaths were expected were placed on the pathway.

### Research question

This study used a qualitative case study design to:

1. Identify factors influencing health care professionals’ decisions to implement the LCP;

2. Understand health care professionals’ experiences of caring for dying cancer patients and how they perceived this care was supported, or not, by the LCP.

## Method

### Study setting

The study was located in three oncology wards representing the in-patient component of a cancer centre located within a large university hospital in England where the lead researcher (AF) is a nurse specialist. There were 204 deaths on the three wards in the year the study was undertaken (median 192, over 3 years).

### Study design

This study used a qualitative case study design with each case representing a patient who had died and their most involved nurse and doctor. In qualitative case study approaches, even though the cases selected may not be generalizable in any statistical sense, a detailed study of their features and context is undertaken to provide insight into pertinent aspects of the wider problem under examination [17]. At the beginning of three consecutive months, April to June 2010, the lead researcher (AF) approached the oncology wards and, with input from the clinical teams, identified the first few patients each month who had died. Any patient known to AF was excluded. A convenience sample of six patients, two per month, with varying gender, age, primary cancer diagnosis, consultant care and use of the LCP were selected. The nurse and doctor who were involved most in the care of the patients in the last days and hours of life were identified and invited to participate in the study by letter.

### Interviews

After obtaining written consent, qualitative, one-to-one, face-to-face interviews were conducted by AF with the healthcare professionals. An aide-memoire of topics was used to guide the interviews. Topics covered included: the experience of the identified patient in their last hours to days of life as perceived by the healthcare professionals; factors that facilitated recognition of dying; factors contributing to the implementation or not of the LCP; the benefits or not of using the LCP; how discussions about dying were conducted with the patient and/or family; and how aspects of caring for dying patients on the cancer wards could be improved.

### Ethical approval

The study was reviewed and approved by the UK National Research Ethics Service and the NHS Hospital Trust Research and Development Department. Data were anonymised. Participant confidentiality was maintained throughout the study. To enhance anonymity when reporting the case studies patient demographics were altered, with care taken to maintain the essence of the situation for each case.

### Data analysis

Interviews were audio-recorded, and then transcribed verbatim. Firstly, the data were scrutinised to construct the story of unfolding dying as experienced by the identified patients, with attention to the implementation or not of the LCP, and how participants described its perceived role or value and the provision of care on a hospital ward. Summary narratives were compiled for each case. The data were then revisited and thematically organised and analysed [18]. This involved developing a thematic framework by identifying key issues and concepts, drawing on both *a priori* issues identified by the researchers and those raised by interviewees themselves. This approach to analysis included initial familiarisation of the transcripts; identification of themes; indexing, in which the transcripts were annotated allocating relevant text to the identified themes; formulation of mind mapping charts and a coding framework table to identify and interpret key and interrelated themes. A brief synopsis of the narratives about the unfolding dying process as reported by participants will be presented for some of the case studies (Cases 1, 2, 3, 4 summarised below). The themes ‘when dying is recognised and care is supported by the LCP’ and ‘when dying is not supported by the LCP’ will be used to present additional data incorporating direct illustrative quotations.

### Case study 1

An elderly man was admitted with bilateral pneumonia: intravenous antibiotics, high flow oxygen, and intravenous fluids were administered. He was confused and disorientated. Although he had a poor chance of survival, the plan for his care, as advised by the consultant oncologist, was to continue supportive care; if he deteriorated the LCP was to be started. Over the next 24 hours his oxygen requirements increased and he became drowsy and unresponsive. The fear and anxiety of the family were recognised:

*Every time he closed his eyes the family was trying to wake him up. The doctor took them to one side and said look this is what is happening…they came back and they were calm, and they said ‘thank you’ and they accepted that he was going to pass away quite quickly…they were relaxed, and it was a nice atmosphere in the room* (Nurse: moderately experienced).

Participants reported that this approach enabled the junior doctor to approach the situation without delay and, with confidence, engage the family in discussion and prepare them for the imminent death. In commencing the LCP the focus became comfort, dignity and good symptom control. Participants reported that the family found clear communication helpful; having a clear plan facilitated comfortable dying. He died two days after admission.

### Case study 2

A young woman with advanced metastatic breast cancer was admitted with deteriorating liver and kidney function. The day after admission, on the ward round, the consultant took the patient’s husband aside and told him that his wife was dying. The patient continued to eat, drink and take oral medications. Over the next few days her condition deteriorated and the LCP was commenced. When too weak to swallow, her physical symptoms including seizures, were controlled using medications in a continuous subcutaneous infusion. She became drowsy and slipped into unconsciousness; she died six days after admission.

Despite directly questioning the consultant: “Am I dying?” the patient was not included in the initial discussion about dying. It was the Macmillan nurse who engaged in these difficult conversations and supported the nurses and junior doctor in continuing to explore the patient’s worries and concerns.

Participants reported that recognition of the dying phase and honest communication was helpful, it provided focused time to help to prepare the patient’s young children for their mother’s death; provided an opportunity to address the patient’s anxiety about the dying process and to explore preferred place of death. She died six days after admission.

### Case study 3

A middle aged man with prostate cancer was admitted with hypercalcaemia. Realising the potential seriousness of the situation and with a son living abroad, the family sought guidance from the junior doctor about whether the son should fly home. When the consultant reviewed the patient the junior doctor was not present so the opportunity to discuss the situation in the context of the family circumstances was missed.

*Maybe if we had as a team sat the family down and said “It’s not looking very good, something is going on, we’re not reversing this, he may not survive this episode of illness” maybe that would have prepared them a bit more* (Nurse: moderately experienced).

The patient’s confusion increased and he became drowsy. On the consultant ward round on Friday, the week-end care plan was formulated which included instigating the LCP if he deteriorated further. This was discussed with the family but the patient died on the Sunday, not on the LCP, and before the son arrived.

Participants reported that daily blood tests, intravenous fluids and regular observations continued. Discussing how things would have been different if the patient was on the LCP the doctor commented:

*I think it’s knowing that it’s OK not to do the obs [observations], literally. I know that sounds really silly but sometimes you kind of need permission to stop doing stuff like that…but I guess just being left in peace really would have been better for him* (Doctor: moderately experienced).

### Case study 4

A young man with lung cancer had been considered for palliative chemotherapy; this had never commenced due to his rapid deterioration. He was admitted with a chest infection which was treated with intravenous antibiotics, fluids and oxygen. A nurse describes his last afternoon (a Sunday):

*On his last day we wheeled him outside, it was a really lovely day …after a couple of hours he took a turn for the worse, he wasn’t responding to us and the nurse looking after him, she was ringing the registrar saying, “Can we get him on the Pathway? Or can you at least come and see him because he has taken a turn for the worse?” And they wouldn’t come and see him* (Nurse: moderately experienced).

The on call doctor had previously prescribed medication for symptom control in accordance with the LCP algorithm but had not communicated with the family about possible deterioration. So although the nursing staff had medication to help his symptoms they struggled with the situation:

*I don’t think they [the family] had a lot of preparation. It would have been nice for the doctor to have spoken to them because he was still having all of his treatment, they can see he’s still having fluids, and we’re giving him antibiotics, we’re checking his blood pressure, so in a way that’s hope for them that he might pull round. We knew that wasn’t going to happen…but in that situation you can’t say “All we want to do is keep him comfortable because this is his last few days” you can’t say that because it’s not really been broached* (Nurse: moderately experienced).

Failure to instigate the LCP was seen to contribute to a situation whereby the nurses felt unable to talk honestly to the family about the reality of the situation and prepare them for the death which occurred later that night.

## Results

Four doctors and seven nurses were interviewed in relation to six patient case studies. No-one refused to participate. For case study 2 neither doctor nor nurse had children themselves and identified this as a factor in their lack of knowledge and confidence with exploring the needs and support offered to a dying parent with young children. Therefore for this case study an additional nurse was recruited to further explore this aspect of care. One doctor left the Trust before an interview could be arranged; inability to arrange a convenient time within the time constraints of the study prevented a further interview with a doctor. Time since qualification of the doctors ranged from 7 months to 4 years and for nurses from 14 months to 22 years. The level of experience of healthcare professionals were combined into groups: the terms ‘inexperienced’ (less than two years), ‘moderately experienced’ (less than ten years) and ‘very experienced’ (more than ten years) were used to describe interviewees (Table 1). The mean duration of the interviews was 31 minutes (range 16–51) for doctors and 36 minutes (range 25–42) for nurses. No senior doctors i.e. consultants or specialist registrars were interviewed.

**Table 1:** The level of experience of healthcare professionals interviewed for each case

**Case study**	**Patient demographics**	**Died with LCP: Yes/No**	**Experience of doctor**	**Experience of registered nurse**	**Other healthcare** **professional**
1.	Elderly man, stomach cancer	Yes	Moderately experienced	Moderately experienced	
2.	Young woman, breast cancer, liver and brain metastases	Yes	Inexperienced	Very experienced	Nurse: very experienced
3.	Middle aged man, prostate cancer	No	Moderately experienced	Moderately experienced	
4.	Young man, lung cancer	No		Moderately experienced	
5.	Elderly woman, malignant melanoma	No	Inexperienced	Very experienced	
6.	Middle aged woman, gallbladder cancer	Yes		Inexperienced	

Four patients died supported by the LCP; two did not. The most obvious factor in this difference was the time of death; both patients whose care was not supported by the LCP died ‘out–of-hours’ at a week-end when senior medical cover was significantly reduced.

### When dying is recognised and care is supported by the LCP

#### Transitions in care are better recognised

It was clear from the accounts of both doctors and nurses that they regarded the oncology setting as one in which an awareness of imminent death was recognised and this was a key factor in using the LCP to support care in the dying phase. Not only was recognising dying essential, having the confidence to voice this opinion was also necessary:

*‘…it’s the recognising it and somebody actually saying “this patient is dying, this is what we are doing”…’* (Doctor: moderately experienced).

Although both junior doctors and nurses saw themselves as separate teams, intra- and inter-professional collaboration was evident. With the exception of one inexperienced nurse, all nurses appeared to be proactive in recognising dying and prompting the doctors to ask for guidance in decision making. This approach was accepted positively by the doctors:

*‘They’re obviously very experienced with oncology patients and they realise these decisions need to be made and they’ll prompt us as juniors to approach the seniors about that’* (Doctor: moderately experienced).

*‘And we can get the best out of the doctors because we can get them to write up what we would like them to have, what we would like to give them to make their last few days as comfortable and as distress free as possible’* (Nurse: very experienced).

### Perception of equitable care delivery

Care of the dying prior to the introduction of the LCP was described as a lottery depending on which consultant was looking after the patient. Use of the LCP was seen as helpful since it was seen as providing equitable evidence-based care and clarity*,* enabling all staff to *‘sing from the same hymn sheet’*:

*‘It does make our job a lot easier…as long as people get on the LCP at the right and appropriate time I think we manage things excellently; patients get a good level of care.’* (Nurse: moderately experienced)

### Proactive approach

All participants made reference to anticipatory prescribing [the practice of doctors writing up prescriptions in advance of a patient’s needs, recommended in the LCP]; this was presented as one of the most helpful aspects of the LCP providing a clear logical approach to good symptom control which in turn facilitated comfortable dying. Moreover, it prevented unnecessary delays in medication administration particularly outside normal working hours. Discontinuation of inappropriate interventions was described by all participants as a positive way to enable natural dying and promote dignity and comfort:

*‘We can stop …wheeling them up to X-ray at the slightest cough and we can be sure that we can give that patient the best possible all-round care in the last days of their life, with dignity’* (Nurse: very experienced).

As illustrated in Case Study 1, early senior medical review with a clear documented plan for management in the event of deterioration and approaching death enabled the clinical team on the ward to feel confident about using the Pathway to support care at the appropriate time.

### Job satisfaction

All the participants expressed the view that as death drew near continuing active treatment was unnecessarily distressing and counter to comfort and dignity with job satisfaction gained from enabling a comfortable death:

*‘I think sometimes as doctors we are always focussing on getting …people better, but then you realise that everyone is going to die… it’s just as rewarding to help someone die comfortably in the way that they want to…’* (Doctor: inexperienced)

### Awareness of the reversibility of the LCP

All the doctors and several nurses shared their experiences of patients who had been taken off the LCP when their condition had improved. This was seen as a positive event:

*‘I think it’s also good to know that the LCP is not the final thing, you can stop it and restart things again; you can actually reverse it if needed’* (Doctor: inexperienced).

### When dying is not supported by the LCP

#### Professionals shortage of time

Consultants and registrars were perceived as having many commitments and responsibilities including multi-disciplinary team meetings, clinics and treatment planning. Consultant ward-rounds varied: some consultants *‘will just pop up randomly’* whereas others had designated times which occurred infrequently (once or twice a week) where the patient was *‘seen briefly, for a snapshot’ -* not long enough to recognise subtle deterioration. Consequently, the clinical assessment and communication skills of healthcare professionals to convince the consultant that a patient was dying were reported as being imperative. Week-end plans particularly concerning possible deterioration were helpful but not always forthcoming:

*‘There are some consultants who will not make those decisions – resus, [resuscitation], for or not for escalation, the LCP; the patient is left in a sort of grey area where we just have to deal with things as they come along’* (Doctor: moderately experienced).

#### LCP – Low priority

The presence of patients receiving curative treatments alongside those entering the dying phase resulted in situations where staff had to balance conflicting priorities. This was particularly pertinent when medical cover was reduced outside normal working hours (see for example, Case study 4):

*‘If there are lots of really sick patients, it gets left in terms of, prioritising what you’ve got to do on that day’* (Doctor: moderately experienced).

*‘It’s at week-ends and nights when you struggle…trying to get somebody at night to come…they’re just reluctant.’* (Nurse: moderately experienced)

*‘It was a week-end, and it was –“if he’s still here, we will review it on Monday and then he’ll get put on the pathway”.’* (Nurse: moderately experienced)

#### Consultant veto

All the participants reflected on the difficulty with inconsistent practice whereby a consultant who *‘doesn’t believe in the LCP’* refused to use it:

*‘A doctor on call came and made that decision [to implement the LCP]. The consultant came and tried to reverse it. He took the LCP paperwork out of the notes and said: “Get rid of it.” She died later that day.’* (Nurse: moderately experienced).

A perception of inconsistency amongst consultant staff in approach to recognising the dying phase and implementation of care supported by the LCP resulted in situations where end-of-life decision making was delayed:

*‘If the consultant is not around, people aren’t willing to make decisions, so we delay, waiting for a decision from a consultant.’* (Nurse: moderately experienced)

In the absence of a consultant decision to commence the LCP examples were given of doctors compromising and prescribing anticipatory medications for the five common symptoms of dying, as identified in the LCP prescribing guidelines, but failing to communicate the uncertainty of the situation to the patient and family (see for example, case study 4).

#### Concern that the LCP induces complacency

Several nurses reported that there was a risk that the four-hour assessment *tick-box approach* induced complacency. One doctor expressed concern that once patients’ care was supported by the LCP their medical daily assessment was not always so thorough:

*‘.You don’t think as hard’* (Doctor: moderately experienced)

Additionally, there was a question over whether a daily review by junior doctors was sufficient:

*‘I think towards the end, if they’re dying, I know some families would like to have seen a consultant more often… to keep them reassured that we have done everything and that the LCP is not a pathway to make them die quicker’* (Doctor: inexperienced).

#### Communication difficulties

Discussions with patients and families were predominantly doctor-led. However, as in case study 2, it was more usual for the senior doctor to talk to the family about death and dying but avoid direct communication with the patient. When clear unambiguous communication with the family did not occur, nurses described feeling unable to initiate the conversation (see for example, case study 4). One inexperienced nurse described how she struggled to use the word ‘dying’. It was easier to use ‘The Pathway’ [LCP] as a euphemism for dying:

*‘…you tell the family about the fact that they’ve been put on the Pathway, that there will be no active treatment but it’s part of keeping them comfortable…I think that’s the only way you put it…*’ (Nurse: inexperienced)

Barriers to effective communication were evident but it was not apparent whether it was patients or healthcare professionals who found this hardest:

*‘Sometimes it is very difficult to talk about sensitive issues, they [patients] feel uncomfortable or they’re scared to talk about it or things get brushed under the carpet’* (Nurse: very experienced)

*‘I think it’s difficult to bring it up, you don’t know whether they want to know or not…you try to come across as friendly and approachable, then hopefully they will know that they can ask you anything’* (Nurse: moderately experienced)

#### Difficulties accepting death

The constant flow of dying patients week after week was described as overwhelming by all participants. Although using the LCP was regarded as helpful, three of the least experienced doctors said it could still be difficult to make the decision to use it, especially when young patients were dying:

*‘It was quite difficult for me to begin with… it was normally the elderly patients we put on the LCP and I didn’t feel as bad but when I suddenly saw someone who was quite young with her daughter – that was difficult.…’* (Doctor: inexperienced)

*‘The area I struggle more is when patients are still being actively treated… they’re given straws to clutch at…one more cycle of chemotherapy…we need to recognise when enough is enough. I go along with it but I’m not happy about it.’* (Nurse: very experienced)

## Discussion

Previous research carried out in acute hospitals has suggested benefits of using the LCP to support care such as discontinuation of inappropriate treatments and improving symptom management [3-6]. Findings of this study support this: once the possibility of imminent death was clarified, healthcare professionals appeared confident in their ability to ensure a comfortable death using the LCP; they found the symptom control algorithms helpful; that the prompts for physical, psychological and spiritual care provided a clear plan; and perceived that communication between healthcare professionals and with families improved.

Nevertheless, participants described how the greater emphasis on communication did not always extend to the patient. This is consistent with results from the third round of the National Care of the Dying Audit –Hospitals in which 42% of conscious patients whose care was supported by the LCP were not aware that they were dying; 61% were given the opportunity to discuss what was important to them; of these, only 58% took up the offer [12]. Murphy, working as Lead Nurse for the LCP, stresses the pivotal importance of communication: the plan of care should be communicated to the patient when possible and appropriate and to the relative or carer without fail [11]. However, an international modified Delphi study exploring issues and needs in end-of-life decision making among palliative care experts from nine countries and a variety of professions identified the need for more evidence-based guidance on ‘optimal strategies for communication with the patient’ (87%) and a comparable statement for ‘communication with relatives’ (83%) [19]. This study highlights the level of challenge involved in providing adequate communication.

In a busy hospital environment deciding that a patient is dying and taking responsibility for the decision to implement care supported by the LCP is challenging. In this study access to timely senior review was regarded as an essential preliminary to this; when the LCP was not used rather than lack of recognition of dying being the cause, it was delayed access to senior review that was often regarded as responsible. As also identified in the national confidential enquiry of deaths in acute hospitals this was particularly pertinent outside normal working hours [20]; at these times significant tensions arose from balancing conflicting priorities and although accepted as important the needs of dying patients sometimes failed to compete with those receiving active treatment. O‘Hara [6] describes concerns that arise at week-ends when on-call teams are covering, but suggested that clear documented weekend care plans ensured no delay in commencing the LCP. This was not always the case in this study as illustrated in Case Study 3.

Anecdotally concern has been expressed that senior doctors are reluctant to start the LCP for fear that the patient’s condition should improve and following this the competence and expertise of the doctor be questioned. However, among the four junior doctors interviewed the potential reversibility of the LCP was a positive factor. Recognising that for a small number of patients it is very difficult to predict accurately when they are dying, version 12 LCP specifically includes a regular senior review to ensure the appropriate continued use of the tool. As a minimum this is recommended every three days.

When the use of the LCP is accepted as hospital policy and experienced as helpful by nurses and junior doctors, outmoded hierarchies in decision making with reluctance and disregard for its implementation by senior doctors, who are essential to the decision to start care supported by the LCP can create tension. Jack et al. reported nurses finding resistance to using the LCP among consultants [3]. It is concerning that six years after implementing the LCP this attitude is still evident.

Disregard for the use of recognised tools to deliver end-of-life care may become increasingly significant as funding from commissioners becomes linked to compliance with use of the LCP. Although, in hindsight all the selected cases examined in this study met the criteria for starting care supported by the LCP, participants were able to identify several other patients for whom they had provided care who had deteriorated very suddenly and died without the LCP. Most commonly, these deaths were reported to be as a result of an unexpected event either during treatment or when coming towards the end of life e.g. pulmonary embolism, haemorrhage or sepsis. This is consistent with observations made by Pugh, McEnvoy and Blenkinsopp who suggest that the LCP may be appropriate for just 80% of patients dying with cancer [21]. The challenge to comply with funding targets from commissioning bodies will be to ensure that for those patients who are expected to die care is supported by the LCP. Identifying potential obstacles to commencing this approach to care at end of life may be helpful. Good end-of-life care is a right for everyone; this study would suggest that use of the LCP is part of that.

### Study limitations

This was a time-limited exploratory study completed as part of a Masters degree. Results need to be interpreted with care as the sample size was small with just six case studies. Furthermore, the study is limited by the absence of experiences of healthcare professionals other than junior doctors and ward nurses. The reality may be that these are the healthcare professionals who are present in the last days and hours of life. Notwithstanding, perspectives of senior medics who are instrumental in taking the decision to start the LCP would add an extra dimension. Additionally, the researcher worked as a nurse specialist in the same hospital where the study took place and had a role in implementing and supporting the LCP, this may have influenced comments. Furthermore, although the research process was discussed, agreed and supervised by the academic supervisor (JS), data analysis was conducted by a single researcher.

## Conclusion

This exploratory study has contributed insights into understanding how a small number of doctors and nurses strive to deliver compassionate care to dying patients on busy hospital wards. Whilst recognition of dying for most cancer patients appears to be straight forward there remain a significant number for whom the LCP is not implemented; this is especially pertinent outside normal working hours. Although the LCP is advocated as best practice, adopted by organisations and is viewed positively by nurses and junior doctors, this does not guarantee the co-operation of all medical consultants and can lead to professional tension. Despite significant improvements in the confidence of healthcare professionals to deliver effective symptom control at the end of life, attributed to the education and implementation associated with the LCP, this does not always translate to effective communication about dying, particularly with the patient. Focussed efforts should be directed at education in communication at end of life, addressing priorities of care especially out of hours, ensuring regular senior review of all dying patients and supporting front line staff.

Further research in regard to those patients whose care is not supported by the LCP is needed. Additionally, exploration of out of hours and week-end use of the LCP to support care, the pressures and conflicts inherent in caring for both curative and palliative patients in the same unit; and the impact of re-assessment on day 3 or earlier as required, as in version 12 LCP warrant further research. Eliciting the views of senior staff would add a helpful dimension.

## Abbreviations

LCP: Liverpool care pathway for the dying patient.

## Competing interests

The authors declare that they have no competing interests.

## Authors’ contributions

AF conceived the study, developed the data collection tools, undertook data collection and conducted the data analysis under the supervision of JS. AF and JS contributed to the design of the study and obtained ethics approval. AF was helped by JS to draft the manuscript. All authors read and approved the final manuscript.
